# Hsa_circ_0000520 overexpression increases CDK2 expression via miR-1296 to facilitate cervical cancer cell proliferation

**DOI:** 10.1186/s12967-021-02953-9

**Published:** 2021-07-20

**Authors:** Qingling Zheng, Jin Zhang, Ting Zhang, Yanxiang Liu, Xiuluan Du, Xin Dai, Donghua Gu

**Affiliations:** 1 Department of Obstetrics and Gynecology, School of Medicine, Huzhou University, Huzhou, 313000 People’s Republic of China; 2 Department of Pathology, Suzhou Science & Technology Town Hospital, No. 1, Lijiang Road, Huqiu District, Suzhou, 215153 Jiangsu People’s Republic of China; 3 Department of Pathology, School of Medicine, Huzhou University, Huzhou, 313000 People’s Republic of China

**Keywords:** Cervical cancer, hsa_circ_0000520, microRNA-1296, Cyclin-dependent kinase 2

## Abstract

**Background:**

Circular RNA (circRNA) has been demonstrated to participate in cervical cancer development. In this study, we analyzed the role of hsa_circ_0000520 in cervical cancer.

**Methods:**

Fifty-two pairs of cervical cancer and adjacent normal tissue samples were collected, and five human cervical cancer cell lines were obtained followed by the detection of hsa_circ_0000520 expression. Nuclear-cytoplasmic isolation and fluorescence in situ hybridization were performed to analyze the subcellular localization of hsa_circ_0000520 while linear RNA was digested by RNase R. Gain- or loss-of function experiments on hsa_circ_0000520 were performed, followed by detection of cell proliferation and cell cycle by EdU, Cell Counting Kit-8, colony formation assay, and flow cytometry respectively.

**Results:**

Hsa_circ_0000520 and cyclin-dependent kinase 2 (CDK2) were highly expressed in cervical cancer tissues. Binding sites between microRNA-1296 (miR-1296) and hsa_circ_0000520 or CDK2 were verified. Antibody to Argonaute 2 (Ago2) could precipitate hsa_circ_0000520, indicating that hsa_circ_0000520 could competitively bind to miR-1296 via Ago2. Silencing hsa_circ_0000520 inhibited cervical cancer cell proliferation and promoted the inhibitory effects of miR-1296 on CDK2, thereby blocking cell cycle progression and promoting apoptosis.

**Conclusion:**

These results support the premise that targeting hsa_circ_0000520 can be a potential approach to combat cervical cancer.

**Supplementary Information:**

The online version contains supplementary material available at 10.1186/s12967-021-02953-9.

## Background

Ranking as the 4th frequently occurring cancer among females worldwide, cervical cancer imposes tremendous disease burden, particularly upon low- or middle-income families [1]. Moreover, cervical cancer is the 2nd most lethal cancer among women associated with poor prognosis and low survival rates [2]. Therefore, the pathogenesis of cervical cancer has been extensively explored, which has demonstrated human papillomavirus infection and genetic mutation as crucial to cervical cancer carcinogenesis [3]. Circular RNA (circRNA), a type of closed-loop structural non-coding RNA molecule, functions as a regulator of gene processes [4]. Our study aims to investigate the role of one specific circRNA, hsa_circ_0000520, and its underlying regulatory mechanism in cervical cancer, attempting to identify a potential therapeutic target for cervical cancer.

In previous studies, circRNAs have been reported as biomarkers for cervical cancer. For instance, circ_0031288 is associated with the migration/invasion, proliferation and growth of cervical cancer cells [5]. Circ_homeodomain interacting protein kinase 3 (circHIPK3) was found to promote the epithelial mesenchymal transition in cervical cancer to accelerate progression of cervical cancer [6]. Circ_0000520 is reported as aberrantly expressed in gastric cancer and hepatocellular carcinoma [7, 8]. However, how hsa_circ_0000520 functions in cervical cancer is still under-studied. To our best knowledge, ours is the first study investigating the correlation of hsa_circ_0000520 with cervical cancer. Additionally, circRNAs are proved to bind to microRNAs (miRNAs) and affect the development of different cancers [9]. For instance, hsa_circ_0005576 was found to regulate the expression of miR-153 in cervical cancer cells [10]. In silico evaluation of the binding relationship between hsa_circ_0000520 and miR-1296 has not yet been reported. miR-1296 is implicated in the apoptosis of cervical cancer cells [11]. Yet the detailed regulatory mechanism of miR-1296 involvement remains largely elusive. As for cyclin-dependent kinase 2 (CDK2), increased expression of CDK2 has been identified in the cervical cancer cell line HeLa as previously reported [12]. Nevertheless, specific upstream mechanisms need to be further explored. Our study explored whether hsa_circ_0000520 can exert certain functions affecting the progression of cervical cancer in association with miR-1296 and CDK2, with intent to identify novel targets for cervical cancer treatment.

## Materials and methods

### Ethical statement

All patients provided signed informed consent and the experimental study protocol was approved by the Ethics Committee of Suzhou Science & Technology Town Hospital. The experimental protocol involving animals was approved by the Animal Ethics Committee of Suzhou Science & Technology Town Hospital.

### Bioinformatics analysis

A microarray dataset GSE102686 was downloaded from the Gene Expression Omnibus (https://www.ncbi.nlm.nih.gov/geo/), which used the GPL19978 platform, and included 5 cervical cancer tissue and paired adjacent tissue samples. Differential analysis was performed to screen differentially expressed circRNA in cervical cancer using the “limma” package in R (http://www.bioconductor.org/packages/release/bioc/html/limma.html) with |logFoldChange|> 1 and *p* value < 0.05 as the threshold.

### Clinical samples and cell culture

Fifty-two pairs of primary cervical cancer tissues and adjacent normal tissues were collected from patients (aged from 34 to 70 years with a mean age of 49.21 ± 10.16 years old) from June 2015 to November 2018 at Suzhou Science & Technology Town Hospital and Huzhou Central Hospital. All patients were diagnosed using pathological examination, and the included cases were not complicated by pelvic inflammatory diseases or immune-related diseases. There were 46 cases of stage I–II and 6 cases of stage IIIa based on the International Federation of Gynecology and Obstetrics criteria.

Cervical cancer cell lines (SiHa, HT-3, Hela, SW756 and ME-180) obtained from the American Type Culture Collection (Rockville, MD, USA) were cultured in Dulbecco's modified Eagle medium (DMEM, HyClone, Thermo Scientific, USA) containing 10% fetal bovine serum (FBS), 100 μg/mL streptomycin and 100 IU/mL penicillin at 37 °C with 5% CO_2_. Cells at 90% confluence were subcultured and the culture medium was removed. The expression of hsa_circ_0000520 in 5 cervical cancer cell lines was determined by reverse transcription quantitative polymerase chain reaction (RT-qPCR), and the two cell lines with the highest expression of hsa_circ_0000520 were screened for subsequent experiments.

### RT-qPCR

Trizol (15596026, Invitrogen, Carlsbad, CA, USA) was applied to extract total RNA, and RNA was reverse transcribed into complementary DNA (cDNA) according to the instructions of PrimeScript RT reagent Kit (RR047A, Takara, Japan). PCR was performed using SYBR Premix EX Taq kit (RR420A, Takara) in a real-time fluorescent qPCR instrument (ABI 7500, ABI, Foster City, CA, USA). Primers (Table S1) for hsa_circ_0000520, ribonuclease P RNA component H1 (RPPH1), miR-1296, CDK2, U6 and glyceraldehyde-3-phosphate dehydrogenase (GAPDH) were synthesized by GeneChem (Shanghai, China). U6 was used as the internal reference gene of miR-1296 whereas other genes were normalized to GAPDH. The relative expression of the products was calculated by the 2^−△△Ct^ method.

### Western blot analysis

Total protein was extracted by radio immunoprecipitation assay (RIPA) lysis buffer (R0010, Solarbio, Beijing, China) containing phenylmethylsulfonyl fluoride, incubated on ice for 30 min and centrifuged at 12,000 r/min at 4 °C for 10 min followed by collection of supernatant. The protein concentration of each sample was determined by bicinchoninic acid kit (23225, Pierce, Boston, MA, USA). A total of 50 μg protein sample was separated by 10% sodium dodecyl sulfate polyacrylamide gel electrophoresis for 2 h and transferred to polyvinylidene fluoride membranes (ISEQ00010, Millipore, Billerica, MA, USA). The membrane was blocked with 5% skimmed milk powder for 2 h and incubated with primary antibodies (Abcam, Cambridge, UK) of rabbit anti-human CDK2 (1: 500, ab194868), CyclinD1 (1: 500, ab61758), P21 (1: 1000, ab227443), P27 (1: 1000, ab75908), B-cell lymphoma-2 (Bcl-2) (1: 500, 59348), Bcl-2 associated protein X (Bax) (1: 500, ab53154) and GAPDH (1: 2500, ab9485) overnight at 4 °C. Horseradish peroxidase labeled goat anti-rabbit immunoglobulin G (IgG) (1: 2000, ab6721, Abcam) secondary antibody was added to the membrane and incubated at room temperature for 1 h. Color was developed by enhanced chemiluminescence reaction solution (WBKLS0100, Millipore). With GAPDH as the internal reference, the relative expression of proteins was presented as the value of the target band to the internal reference band.

### Dual-luciferase reporter gene assay

Circ_0000520 transcripts containing the putative miR-1296 binding sites as hsa_circ_0000520-wild type (Wt) and hsa_circ_0000520-Wt mutated at the putative miR-1296 binding sites as hsa_circ_0000520-mutant type (Mut) were inserted into the luciferase reporter vector, respectively. CDK2-Wt and CDK2-Mut were developed similarly. Well-designed reporter vectors were delivered into 293T cells with miR-1296 mimic, respectively. The luminescence of firefly luciferase was determined using dual-luciferase reporter assay system kit (K801-200, BioVision, USA) and normalized to that of renilla luciferase detected following the instructions of Glomax20/20 luminometer (Promega, USA).

### Immunohistochemistry (IHC)

Primary cervical cancer tissues and the matched adjacent normal tissues were fixed with 4% neutral formaldehyde buffer (DF0113, Solarbio), paraffin-embedded, and sectioned at a thickness of 4 μm. The sections were dewaxed with xylene (YB-5485, YBi, Shanghai, China), hydrated with gradient alcohol, and soaked in 3% hydrogen peroxide for 20 min at room temperature to remove endogenous peroxidase activity. Meanwhile, the antigen was thermally retrieved twice and sections were blocked with 10% goat serum for 15 min, which were incubated with rabbit anti-CDK2 primary antibody (1: 500, ab194868, Abcam) overnight at 4 °C. Biotin-labeled goat anti-rabbit IgG (1: 1000, ab6721, Abcam) secondary antibody working solution was added into the sections for 40-min incubation at 37 °C. Color was developed with diaminobenzidine (DA1010, Solarbio) for 10 min, followed by the counterstaining of sections with hematoxylin (H8070, Solarbio) for 1 min. Phosphate buffer saline (PBS) was used instead of primary antibody as negative control (NC). The final results were scored by two pathologists in a blinded-manner. Sections were observed under a light microscope (CX41-12C02, Olympus, Japan) with 5 visual fields randomly selected. The positive cells were seen as brown-yellow. The percentage of positive cells among the total cells indicated CDK2 protein staining results with > 10% considered as positive expression (+) and < 10% as negative expression (−).

### RNase R linear RNA digestion experiment

Cells were seeded into a 6-well plate (2 × 10^5^ cells/well), trypsinized and centrifuged at 2000×*g* for 2 min. Cytoplasm and nuclear RNA were each recovered using a nuclear-cytoplasm separation kit according to standard instructions. RNA was divided into RNase digestion group and non-digestion group. Linear RNA was digested by 10 × reaction buffer with 1 μg RNA digested by 1 unit (U) RNase at 37 °C for 10 min. Subsequently, the digested products were extracted by phenol/chloroform and ethanol precipitation, and reverse transcribed into cDNA.

### RNA fluorescence in situ hybridization (FISH)

Cell slides were cultured at the bottom of the 24-well plate (6 × 10^4^/well). FISH assay was performed when cell confluence reached 60–70%. Cells were fixed with 4% paraformaldehyde for 10 min at room temperature, added with 1 mL of precooled permeate each well and stood at 4 °C for 5 min. Furthermore, each well was blocked with 20 μL pre-hybridization solution for 30 min at 37 °C with hybridization solution preheated at 37 °C simultaneously. After removal of pre-hybridization solution, each well was hybridized with hybridization solution containing probes overnight at 37 °C in the dark. Slides were then stained with 4',6-diamidino-2-phenylindole staining solution for 10 min and mounted for fluorescence detection.

### Cell transfection

Human cervical cancer cells were transfected with short hairpin RNA targeting hsa_circ_0000520 (sh-hsa_circ_0000520), sh-CDK2, CDK2 overexpression vector (oe-CDK2), miR-1296 mimic, miR-1296 inhibitor or relevant NC alone or in combination. All plasmids were purchased from Dharmacon (Lafayette, CO, USA). Cervical cancer cells were seeded in 6-well plates at a density of 3 × 10^5^ cells/well and transfected with the aforesaid plasmids following the instructions of Lipofectamin 2000 kit (Invitrogen). The culture medium was renewed with complete culture medium 6 h post transfection. Cells were further cultured at 37 °C in 5% CO_2_ and then collected after 48 h.

### Colony formation assay

Cells were detached with 0.25% trypsin, triturated into single cells, and suspended in DMEM containing 10% FBS. The cell suspension was diluted and cultured in a dish with 37 °C preheated culture solution at the gradient density of 50, 100, or 200 cells/dish, respectively. After 2–3 weeks of culture, the supernatant was discarded when clones were visible. A total of 5 mL cells were fixed with 4% paraformaldehyde for 15 min and stained with GIMSA’s staining solution for 10–30 min. Clones were counted directly.

### Cell counting kit-8 (CCK-8) assay

After a single cell suspension was prepared, cells were seeded into 96-well plates with a volume of 200 μL cell suspension per well and incubated in an incubator with 6 repeated holes. The plates were removed at 24 h, 48 h and 72 h during incubation, followed by the addition of 10 μL CCK-8 solution (Sigma) and another 2 h-incubation. The absorbance values of each well were measured at 570 nm by enzyme-linked immunoassay (NYW-96 M, NYAW Instrument, Beijing, China). A cell viability curve was plotted with time points as the X-axis and optical density (OD) value as the Y-axis.

### 5-ethynyl-2′-deoxyuridine (EdU) assay

Cells in the logarithmic growth phase were seeded into 96-well plates (5 × 10^4^ cells/well). Each well was incubated with 50 μmol/L EdU medium (a total of 500 μL) for 2 h. Plates were then fixed with 40 g/L paraformaldehyde for 20 min, incubated with 2 mg/mL glycine for 10 min, and permeabilized with 500 μL 0.5% TritonX each well. Cells were further incubated with Apollo staining reaction solution for 30 min in the dark and then Hoechst 33,342 reaction solution for 30 min in conditions void of light. After washing with 0.5% Triton twice, the cells were observed under an inverted fluorescence microscope with the number of cells counted using Image-Pro Plus 6.0 Professional Image Analysis Software (IPP, Texas, USA).

### RNA binding protein immunoprecipitation (RIP) assay

Cells were lysed in an ice bath with RIPA lysis buffer (P0013B, Beyotime Biotechnology, Shanghai, China) for 5 min, and centrifuged at 14,000 rpm for 10 min at 4 °C to remove the supernatant. One part of the cell extract was taken as input whereas the other part was incubated with antibody for co-precipitation. The antibodies used for RIP involved rabbit anti-Argonaute 2 (Ago2) (1: 50, ab32381, Abcam) with IgG antibody as NC.

### Flow cytometry

The density of trypsinized cells was adjusted to 3 × 10^5^ cells/mL, which were seeded in 6-well plates and cultured for 48 h. The single cell suspension was centrifuged at 1000 r.min^−1^ × 5 min followed by removal of the supernatant. Cells were fixed with 70% ice ethanol solution at − 20 °C or 1 h and then added with 20 μL RNA enzyme and reacted at 37 °C for 30 min. Subsequently, cells were incubated with 400 μL propidium iodide (PI) (C0080, Solarbio) for 15 min in the dark, on ice. The cell cycle was detected by a flow cytometer (BDLSR II, BD, FL, NJ, USA) with the excitation light wavelength at 488 nm. For detection of cell apoptosis, the cells were resuspended with 400 μL of Annexin V binding solution (CA1020, Solarbio) to 3 × 10^5^ cells/mL and then incubated with 50 μL Annexin V-Fluorescein Isothiocyanate staining solution on ice, in the dark, for 15 min. After the addition of 10 μL PI staining solution, cell apoptosis was detected by flow cytometer also with the excitation light wavelength as 488 nm.

### Xenograft tumor formation in nude mice

Thirty-two BALB/C nude mice (aged 4 weeks and weighed 18–22 g; Animal Experimental Center of Southern Medical University) were treated with SiHa cells overexpressing CDK2 (by transfection of oe-CDK2) and/or silencing miR-1296 (by transfection of miR-1296 inhibitor) or hsa_circ_0000520 (by transfection of sh-circ_000052) with eight mice in each group. Specifically, SiHa cells were prepared into a single cell suspension by suspension in the mixture (PBS: Matrigel = 1: 1) with the final cell concentration as 1 × 10^6^ cells/200 μL. Additionally, nude mice were anesthetized with sodium pentobarbital and injected with SiHa cells subcutaneously at the back of the right hind leg, and were raised in the same environment as the untreated mice. Tumor volume was monitored once a week. At the end of the fourth week, the nude mice were euthanized and tumors were removed.

### Statistical analysis

SPSS 21.0 software (IBM Inc., Armonk, NY, USA) was used for statistical processing of the measurement data derived from experiments. All experiments were repeated at least three times independently. Measurement data were presented as mean ± standard deviation. Cervical cancer tissues were compared with adjacent normal tissues using paired *t* test. Independent sample *t*-test was used for comparison between two groups, whereas one-way analysis of variance (ANOVA) was applied to data comparison between multiple groups followed by Tukey’s post hoc test. Data at different time points were analyzed using repeated measures ANOVA followed by Bonferroni’s post hoc test. The statistical significance was set at *p* < 0.05.

## Results

### Hsa_circ_0000520 was highly expressed in cervical cancer cells

**Fig. 1:**
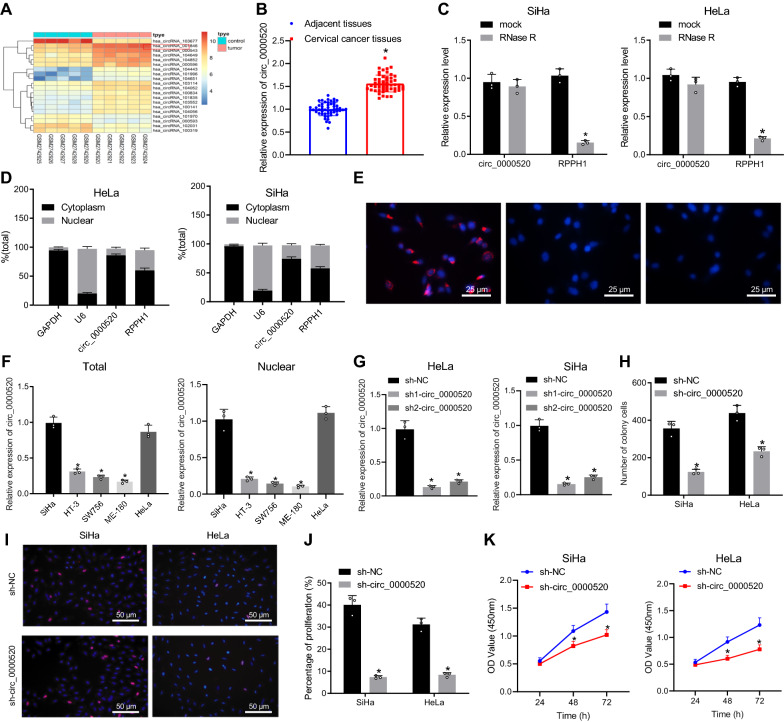
Hsa_circ_0000520 is highly expressed in cervical cancer. **A** Heatmap displaying the expression of top 20 differentially expressed circRNAs in the microarray dataset GSE102686. **B** Expression of hsa_circ_0000520 determined in clinical cervical cancer tissues (n = 52) by RT-qPCR. **C** Resistance of hsa_circ_0000520 to RNase R digestion verified by RNase R linear RNA digestion experiment with RT-qPCR. **D** Content of hsa_circ_0000520 in nuclei normalized to U6 (nuclear control transcript), and cytoplasm normalized to GAPDH (cytoplasmic control transcript) measured by nuclear-cytoplasm isolation assay. **E** Subcellular localization of hsa_circ_0000520 by FISH assay. **F** Expression of hsa_circ_0000520 in different cervical cancer cell lines and in the nucleus after nuclear-cytoplasm isolation assay. **G** Silencing efficiency of sh1-hsa_circ_0000520 and sh2-hsa_circ_0000520 determined by RT-qPCR. **H** Number of cell clones in each group. **I** Images of cell proliferation by EdU assay. **J** Percentage of proliferation. **K** Effects of hsa_circ_0000520 on proliferation of cells determined using CCK-8 assay. *p < 0.05 *vs.* adjacent normal tissues, RPPHI, cytoplasm, SiHa cells or cells in the sh-NC group. Measurement data are presented as mean ± standard deviation derived from at least 3 independent experiments. Cancerous tissues are compared with adjacent normal tissues using paired *t*-test. Data from two unpaired groups are compared by independent sample *t* test. Data from multiple groups are compared by one-way ANOVA followed by Tukey’s test. Data at different time points are compared by repeated measures ANOVA followed by Bonferroni’s test

To predict the role of hsa_circ_0000520 in cervical cancer, bioinformatics analysis was performed. As the analysis of the cervical cancer-related microarray GSE102686 indicated, hsa_circ_001846 was highly expressed in cervical cancer (Fig. 1A). According to circBase (http://circrna.org/), circRNA ID of hsa_circ_001846 is hsa_circ_0000520, that has been reported to promote the occurrence and development of gastric cancer [13]. To verify the results of bioinformatics analysis, RT-qPCR was performed on 52 pairs of cervical cancer tissues and adjacent normal tissues collected from clinical specimens, showing that hsa_circ_0000520 expression was higher in cervical cancer tissues than adjacent normal tissues (Fig. 1B). The resistance of hsa_circ_0000520 to RNase R digestion was verified by RNase R linear RNA digestion experiment, the results of which displayed that hsa_circ_0000520 was generated by RPPH1 transcript alternative splicing. Therefore, we used linear RPPH1 as a control. The RT-qPCR results showed that expression of hsa_circ_0000520 did not change significantly before and after RNase R treatment, but the expression of linear RPPH1 decreased (Fig. 1C). Nuclear-cytoplasm isolation and FISH assays revealed that hsa_circ_0000520 was mainly expressed in the cytoplasm (Fig. 1D, E). Additionally, hsa_circ_0000520 expression in cell lines HT-3, SW756, and ME-180 was lower than that in SiHa and HeLa cell lines (Fig. 1F). hsa_circ_0000520 expression following nuclear-cytoplasm isolation was significantly reduced in the nucleus of HT-3, SW756, and ME-180 cell lines than that in SiHa and HeLa cell lines. Therefore, SiHa and HeLa cell lines were selected for subsequent study. Expression of hsa_circ_0000520 was silenced in SiHa and HeLa cells by delivering sh1-hsa_circ_0000520 and sh2-hsa_circ_0000520. The RT-qPCR results showed that hsa_circ_0000520 expression was lower after treatment with sh1-hsa_circ_0000520, so sh1-hsa_circ_0000520 was selected for further silencing of hsa_circ_0000520 (Fig. 1G). Results of the clone formation assay showed that the amount of cell clones was reduced when hsa_circ_0000520 was silenced (Fig. 1H). Moreover, results of EdU and CCK-8 assays showed that compared with the sh-NC group, cell proliferation was distinctly inhibited by sh-hsa_circ_0000520 (Fig. 1I–K). Taken together, the experimental data demonstrated high expression of hsa_circ_0000520 in cervical cancer.

### Hsa_circ_0000520 promoted the proliferation of cervical cancer cells

**Fig. 2:**
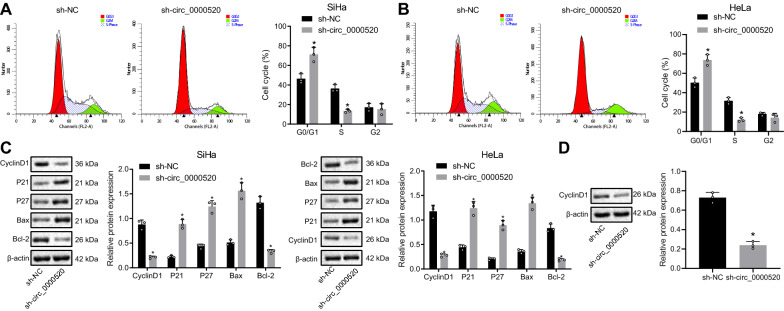
Hsa_circ_0000520 promotes cervical cancer cell cycle. **A** Effects of hsa_circ_0000520 on cycle distribution of SiHa cells evaluated by flow cytometry. **B** Effects of hsa_circ_0000520 on cycle distribution of HeLa cells evaluated by flow cytometry. **C** Levels of cycle-related and apoptosis-related proteins determined by Western blot analysis after hsa_circ_0000520 was silenced. **D** CyclinD1 expression mediated by hsa_circ_0000520 in the nucleus of cervical cancer cells detected by Western blot analysis. *p < 0.05 vs*.* the sh-NC group. Measurement data are presented as mean ± standard deviation derived from at least 3 independent experiments. Data from two groups are compared by independent sample *t* test

Afterwards, the effects of hsa_circ_0000520 on the cell cycle distribution was determined by flow cytometry, which showed that silenced hsa_circ_0000520 arrested more cells in G0/G1 phase and fewer cells in S phase (Fig. 2A, B). Moreover, protein levels of cell cycle-related factors CyclinD1, P21, and P27 as well as apoptosis-related genes Bax and Bcl-2 were detected by Western blot analysis, which displayed increased levels of P21, P27, and Bax after hsa_circ_0000520 expression was silenced, while levels of CyclinD1 and Bcl-2 decreased (Fig. 2C). After further nucleocytoplasmic separation, the downregulation of CyclinD1 was also observed in presence of sh-hsa_circ_0000520 as compared with sh-NC (Fig. 2D). These findings indicated that hsa_circ_0000520 mediated the cell cycle distribution of cervical cancer cells.

### Hsa_circ_0000520 inhibited the expression of miR-1296

**Fig. 3:**
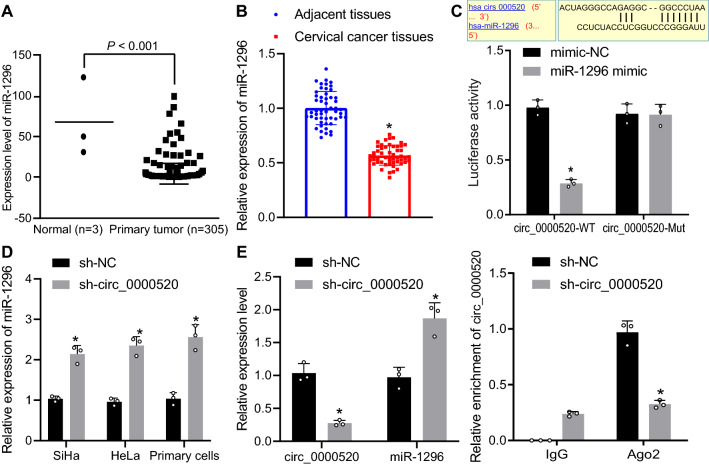
Hsa_circ_0000520 targets miR-1296. **A** Results of TCGA database to analyze the expression of miR-1296 in cervical cancer tissues. **B** RT-qPCR to detect the expression of miR-1296 in cervical cancer tissues and adjacent normal tissues (n = 52). **C** Binding relation between hsa_circ_0000520 and miR-1296 predicted using a web-based tool and verified by dual luciferase reporter gene assay. **D** Expression of mir-1296 detected by RT-qPCR after hsa_circ_0000520 silencing. E, Binding of hsa_circ_0000520 to Ago2 proteins assessed by RIP. *p < 0.05 vs*.* adjacent normal tissues, sh-NC, mimic NC or IgG. Measurement data are presented as mean ± standard deviation derived from at least 3 independent experiments. Cancerous tissues are compared with adjacent normal tissues using paired *t*-test. Data from two groups are compared by independent sample *t* test

To investigate the downstream mechanism of hsa_circ_0000520, the TCGA database was employed to predict the expression levels of miR-1296, which showed that the miR-1296 was underexpressed in cervical cancer (Fig. 3A). The results of RT-qPCR indicated that miR-1296 expression was reduced in cervical cancer tissues compared with adjacent normal tissues (Fig. 3B). Using an online prediction tool it was revealed that there existed binding sites between hsa_circ_0000520 and miR-1296, and the results of dual luciferase reporter gene assay showed that the luminescence decreased in the WT miR-1296 mimic group (Fig. 3C). RT-qPCR analysis further showed that the expression of miR-1296 increased after knockdown of hsa_circ_0000520 in cervical cancer cell lines while miR-1296 was also elevated in cervical cancer primary cells with hsa_circ_0000520 knocked down (Fig. 3D), indicating that hsa_circ_0000520 can specifically bind to miR-1296.

Whether hsa_circ_0000520 could directly interact with Ago2 proteins was assessed by RIP assay. The results showed that sh-hsa_circ_0000520 significantly decreased hsa_circ_0000520 expression and increased miR-1296 expression while anti-Ago2 could precipitate hsa_circ_0000520 (Fig. 3E). Moreover, hsa_circ_0000520 that bound to Ago2 was reduced by sh-hsa_circ_0000520, indicating that hsa_circ_0000520 could form a complex with Ago2 and further suggested that hsa_circ_0000520 could bind to miR-1296.

### Hsa_circ_0000520 promoted the CDK2 expression by binding to miR-1296

**Fig. 4:**
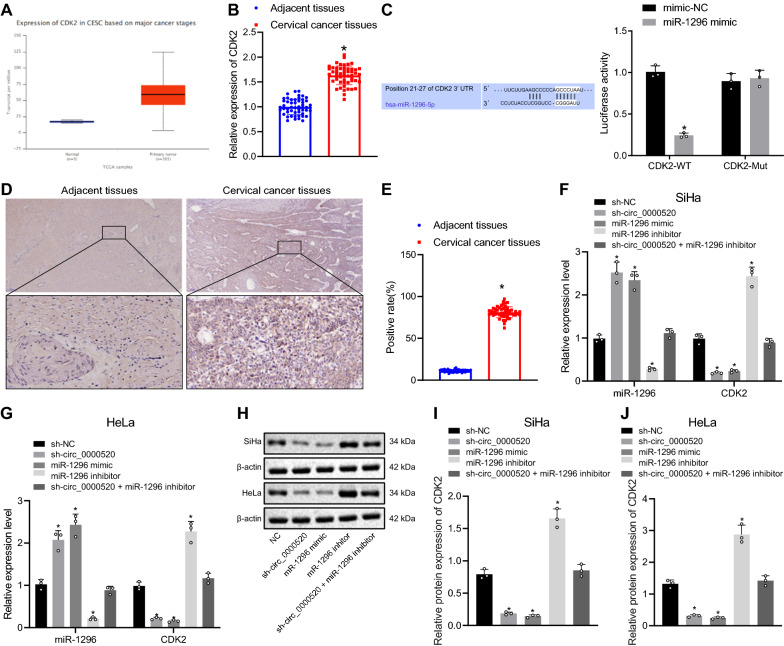
Hsa_circ_0000520 promotes CDK2 expression by suppressing miR-1296 expression. **A** TCGA database analysis results of CDK2 expression in cervical cancer tissues. **B** CDK2 expression in clinical specimens (n = 52) determined by RT-qPCR. **C** Binding relation between miR-1296 and CDK2 predicted using a web-based tool and verified by dual luciferase reporter gene assay. **D** IHC results of CDK2 expression (×200). **E** CDK2 protein expression positive rate. **F** Expression of miR-1296 and CDK2 in each group of SiHa cells determined by RT-qPCR. **G** Expression of miR-1296 and CDK2 in each group of HeLa cells determined by RT-qPCR. **H** Protein bands of CDK2 detected by Western blot analysis. **I** Quantitative analysis of CDK2 expression in SiHa cells determined by Western blot analysis. **J** Quantitative analysis of CDK2 expression in HeLa cells determined by Western blot analysis. *p < 0.05 vs. adjacent normal tissues, mimic NC or sh-NC. Measurement data are presented as mean ± standard deviation derived from at least 3 independent experiments. Data from two groups are compared by independent sample *t* test. Data from multiple groups are compared by one-way ANOVA followed by Tukey’s test

TCGA database further demonstrated that CDK2 was highly expressed in cervical cancer (Fig. 4A). Results of RT-qPCR revealed that the expression of CDK2 was increased in cervical cancer tissues compared with adjacent normal tissues (Fig. 4B). Using prediction software circMir, it was revealed that CDK2 was a potential target of miR-1296 along with 6 bp binding sites between CDK2 3’-UTR and miR-1296 (Fig. 4C). The results of dual luciferase reporter gene assay showed that the luminescence of WT miR-1296 mimic decreased, which indicated that miR-1296 specifically bound to CDK2. Moreover, IHC results showed that CDK2 was mainly expressed in the cytoplasm, and the positive cells were shown in brown-yellow (Fig. 4D). It was also shown that the positive expression rate of CDK2 in adjacent normal tissues was significantly lower than that in cervical cancer tissues (Fig. 4E).

The results of RT-qPCR and Western blot analysis showed that miR-1296 was downregulated in the cells transfected with miR-1296 inhibitor, while the expression of CDK2 increased. Moreover, miR-1296 was upregulated in the cells transfected with miR-1296 mimic or sh-hsa_circ_0000520, along with downregulated CDK2. Expression of miR-1296 and CDK2 did not significantly change in cells transfected with both sh-hsa_circ_0000520 + miR-1296 inhibitor (Fig. 4F–I).

### Silencing of hsa_circ_0000520 inhibited cervical cancer cell proliferation via miR-1296

**Fig. 5:**
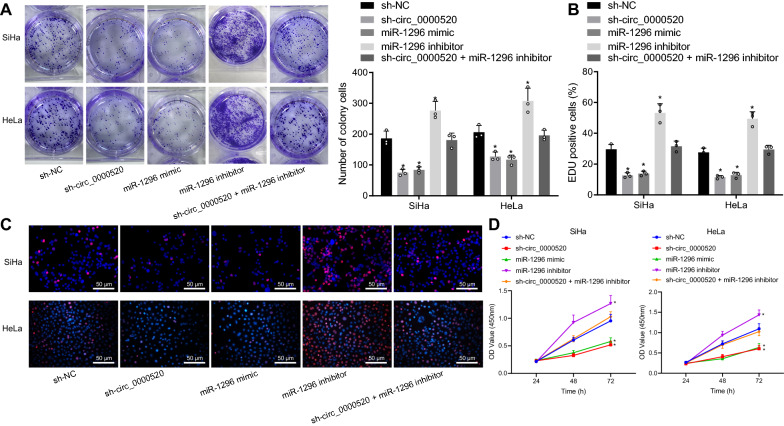
Proliferation of cervical cancer cells is suppressed by overexpressed miR-1296. **A** Results of colony formation assay following altered expression of miR-1296. **B** Cell proliferation rates of cells with altered expression of miR-1296 assessed by EdU assay. **C** Fluorogram displaying cell proliferation detected by EdU assay. **D** OD value of cells in each group determined by CCK-8 assay. *p < 0.05 vs*.* sh-NC. Data from multiple groups are compared by one-way ANOVA followed by Tukey’s test. Measurement data are presented as mean ± standard deviation derived from at least 3 independent experiments. Data at different time points are compared by repeated measures ANOVA followed by Bonferroni’s test

To further validate that hsa_circ_0000520 promoted cervical cancer cell proliferation and cycle distribution by targeting and inhibiting miR-1296, colony formation, EdU and CCK-8 assays were performed. It was shown that the number of cell clones reduced in cells transfected with sh-hsa_circ_0000520 or miR-1296 mimic. However the opposite results were found in cells transfected with miR-1296 inhibitor (Fig. 5A). Moreover, cell proliferation was inhibited by miR-1296 mimic or sh-hsa_circ_0000520, but increased by miR-1296 inhibitor (Fig. 5B–D).

**Fig. 6:**
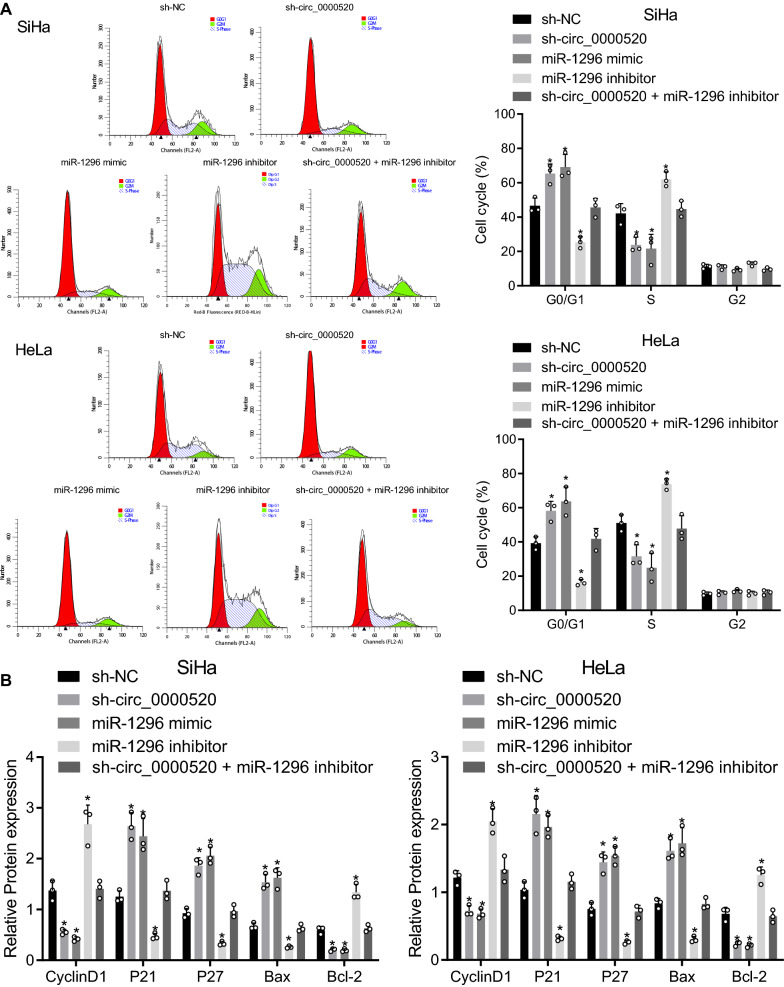
Cell cycle progression is blocked by overexpressed miR-1296. **A** Cell cycle distribution of cells in each group determined using flow cytometry. **B** Cell cycle-related and apoptosis-related protein levels detected using Western blot analysis. *p < 0.05 vs. sh-NC. Measurement data are presented as mean ± standard deviation derived from at least 3 independent experiments. Data from multiple groups are compared by one-way ANOVA followed by Tukey’s test

Flow cytometry results further exhibited that overexpressed miR-1296 or silenced hsa_circ_0000520 arrested more cells in G0/G1 phase and fewer cells in S phase, but miR-1296 inhibitor produced the contrary results (Fig. 6A). Results of Western blot analysis showed that levels of P21, P27, and Bax increased in cells transfected with miR-1296 mimic or hsa_circ_0000520, accompanied with reduced levels of CyclinD1 and Bcl-2. Moreover, after miR-1296 inhibitor treatment, the levels of P21, P27, and Bax decreased, and levels of CyclinD1 and Bcl-2 were enhanced (Fig. 6B).

### Hsa_circ_0000520 knockdown suppressed cervical cancer cell proliferation by downregulating CDK2 via miR-1296

**Fig. 7:**
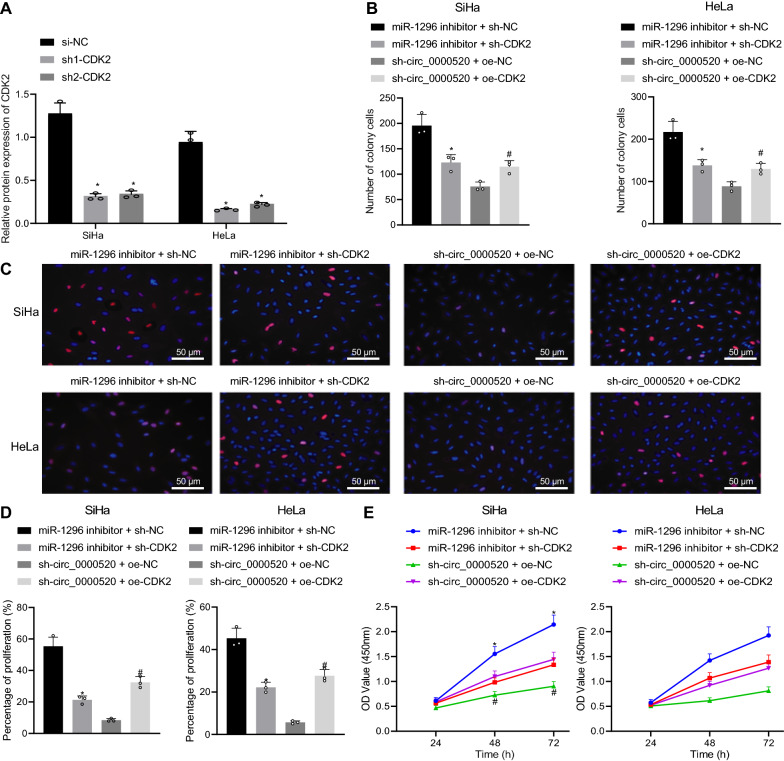
Cell proliferation can be suppressed by silenced CDK2. **A** CDK2 expression detected by Western blot analysis. **B** Results of colony formation assay on cells in each group. **C** Images of cell proliferation determined by EdU assay. **D** Cell proliferation rate assayed by EdU assay. **E** OD values of cells in each group measured by CCK-8 assay. *p < 0.05 vs. sh-NC or miR-1296 inhibitor + sh-NC. ^#^*p* < 0.05 vs. sh-hsa_circ_0000520 + oe-NC. Measurement data are presented as mean ± standard deviation derived from at least 3 independent experiments. Data among multiple groups are compared by one-way ANOVA followed by Tukey’s test. Data at different time points are compared by repeated measures ANOVA followed by Bonferroni’s test

In order to prove that hsa_circ_0000520-mediated miR-1296 inhibition could promote cell proliferation and cell cycle progression by upregulating CDK2, CDK2 expression was suppressed by sh1-CDK2, which showed the best silencing efficiency as Western blot analysis revealed (Fig. 7A). Colony formation assay revealed that the number of cell clones was reduced after cells were co-transfected with both miR-1296 inhibitor and sh-CDK2, which was then increased after cells were co-transfected with both sh-hsa_circ_0000520 and oe-CDK2 (Fig. 7B). In addition, the results of EdU and CCK-8 assays showed that proliferation of cervical cancer cells was suppressed by both miR-1296 inhibitor and sh-CDK2. However, cell proliferation was enhanced by the combined treatment of sh-hsa_circ_0000520 and oe-CDK2 (Fig. 7C–F).

**Fig. 8:**
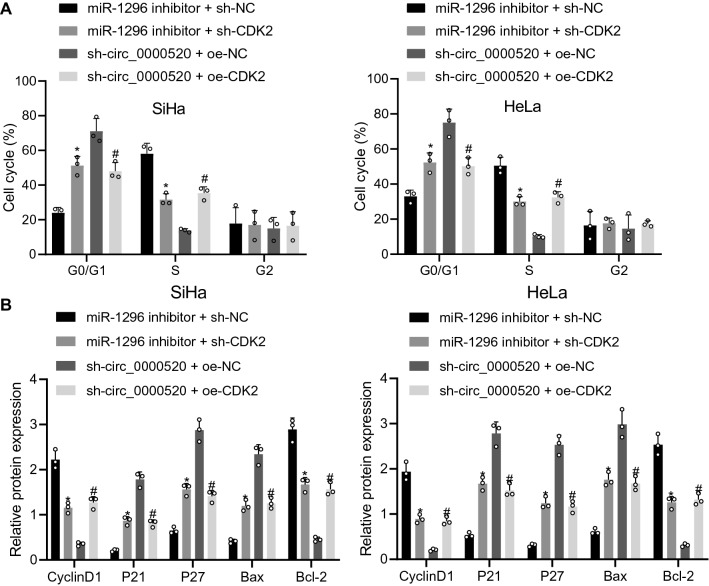
Cell cycle progression can be hindered by depleted CDK2. **A** Cell cycle progression of each group of cells determined using flow cytometry. **B** Levels of cycle-related and apoptosis-related proteins determined by Western blot analysis. *p < 0.05 vs*.* miR-1296 + sh-NC. ^#^*p* < 0.05 vs*.* sh-hsa_circ_0000520 + oe-NC. Measurement data are presented as mean ± standard deviation derived from at least 3 independent experiments. Data among multiple groups are compared by one-way ANOVA followed by Tukey’s test

Moreover, flow cytometry revealed that simultaneous inhibition of miR-1296 and CDK2 arrested more cells in G0/G1 phase and fewer cells in the S phase. However, inhibited hsa_circ_0000520 and overexpressed CDK2 induced the opposite effects (Fig. 8A). As Western blot analysis results showed, the levels of P21, P27 and Bax was elevated in the cells co-transfected with miR-1296 inhibitor and sh-CDK2, while the levels of CyclinD1 and Bcl-2 were reduced. However, levels of P21, P27 and Bax were reduced in cells co-transfected with both sh-hsa_circ_0000520 and oe-CDK2, along with enhanced levels of CyclinD1 and Bcl-2 (Fig. 8B).

### Hsa_circ_0000520 promoted the tumorigenesis of human cervical cancer cells through the miR-1296/CDK2 axis in vivo

**Fig. 9:**
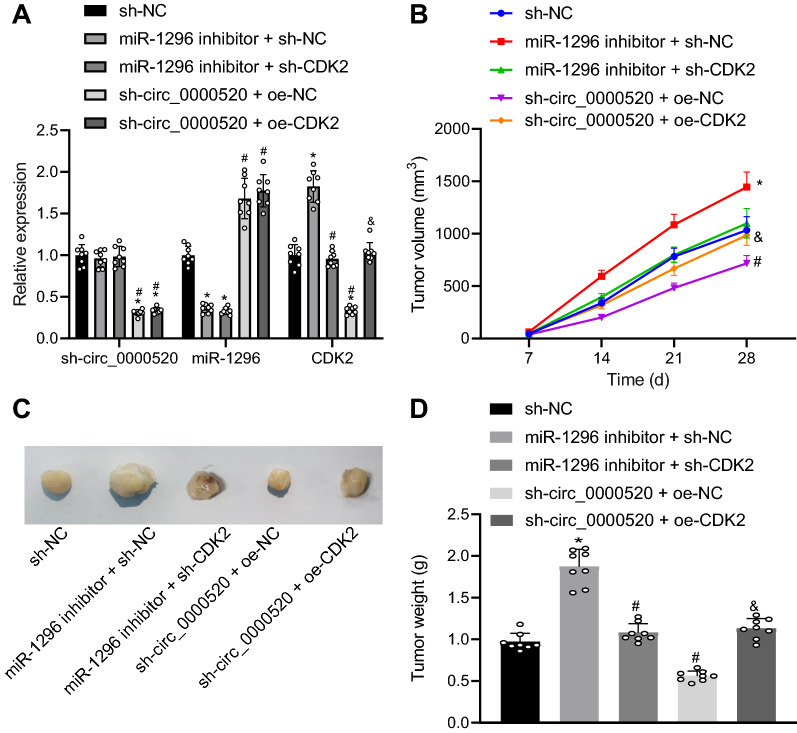
Impacts of hsa_circ_0000520/miR-1296/CDK2 on tumorigenesis in vivo*.* **A** Expression of hsa_circ_0000520, miR-1296 and CDK2 determined by RT-qPCR. **B** Tumor volume in differently treated mice (n = 8/each group). **C** Images of tumors in differently treated mice. **D** Tumor weight in differently treated mice (n = 8/each group). *p < 0.05 vs*.* mice injected with sh-NC-treated cells. ^#^*p* < 0.05 vs*.* mice injected with miR-1296 inhibitor + sh-NC treated cells. ^&^*p* < 0.05 vs*.* mice injected with cells transfected with sh-hsa_circ_0000520 + oe-NC. Measurement data are presented as mean ± standard deviation derived from at least 3 independent experiments. Data among multiple groups are compared by one-way ANOVA followed by Tukey’s test. Data at different time points are compared by repeated measures ANOVA followed by Bonferroni’s test

Finally, to study the function of hsa_circ_0000520/miR-1296/CDK2 in cervical cancer in vivo, tumorigenesis in nude mice was developed. The expression levels of hsa_circ_0000520, miR-1296 and CDK2 were verified and the results confirmed the successful silencing or overexpression (Fig. 9A). The volume and weight of tumors were found increased in the mice injected with cells harboring miR-1296 inhibitor but reduced in those with sh-hsa_circ_0000520. In presence of miR-1296 inhibitor, further treatment with sh-CDK2 resulted in lower volume and weight of tumors. However, the volume and weight of tumors was increased in the mice subjected to co-treatment with sh-hsa_circ_0000520 and oe-CDK2 when compared with sh-hsa_circ_0000520 treatment alone (Fig. 9B–D). These results indicate that silencing of hsa_circ_0000520 or overexpressing miR-1296 reduced the weight and volume of graft tumors in nude mice with cervical cancer.

## Discussion

circRNAs are reported to bind to miRNAs, which can change the translation of target mRNAs and further mediate gene expression at the transcriptional level [14, 15]. Although the circRNA-miRNA-target gene interaction network has been utilized to detect the radioresistance of cervical cancer cell line HeLa [16], more detailed analyses of regulatory networks involving circRNA-miRNA in cervical cancer progression have not been discussed. As a consequence, in this study we focused on the role of hsa_circ_0000520 in both cervical cancer tissues and cells, aiming to lay a theoretical premise for studies focused on cervical cancer therapy. Collectively, the experimental data in our study identified the high expression of hsa_circ_0000520 in both cervical cancer tissues and cells, yet silencing of hsa_circ_0000520 led to suppressed malignant cell proliferation and induced cell apoptosis, and the underlying mechanism was revealed to be dependent on miR-1296 upregulation and CDK2 downregulation.

We showed that hsa_circ_0000520 was highly expressed in cervical cancer tissues and cells. High expression of circRNAs is commonly detected in cancers, such as circ_0018289 and circ-0033550 in cervical cancer [17, 18]. However, the expression pattern of hsa_circ_0000520 in cervical cancer has not been addressed previously. The evidence in the present study further showed that hsa_circ_0000520 promoted the proliferation of cervical cancer cells and accelerated the cell cycle progression. Similarly, circ_000284 activation also enhances the invasion and proliferation of cervical cancer cells [19], which also supports that circRNAs are implicated in cervical cancer development. Moreover, we found that depleted hsa_circ_0000520 induced increased expression of P21 and P27 but decreased CyclinD1 expression. As previously reported, CyclinD1 is considered as one of the cell-cycle inducers while cell-cycle inhibitors include P21 and P27 [20]. Therefore, it may be inferred that silencing of hsa_circ_0000520 was able to block the progression of cell cycle in cervical cancer.

Moreover, both bioinformatic analysis and dual-luciferase reporter gene assay revealed the targeting relationship between hsa_circ_0000520 and miR-1296, showing that hsa_circ_0000520 targeted miR-1296. To our best knowledge, the relationship between hsa_circ_0000520 and miR-1296 was largely unexplored despite increasing evidence that has reported the regulatory mechanisms underlying circRNA and miRNA roles in cervical cancer. For instance, circ_000596-miR-15b and circ_101958-miR-106b regulatory axes are found in cervical cancer [21]. Our study further addressed the research gap concerning the role of circRNA-miRNA networks in cervical cancer. Moreover, we found that miR-1296 expression could be suppressed by overexpressed hsa_circ_0000520. Likewise, in cervical cancer HeLa cells, downregulated circ_0031288 led to upregulation of miR-139-3p [5]. Subsequently, we proved that hsa_circ_0000520-mediated inhibition of miR-1296 was able to promote CDK2 expression in cervical cancer. It has been shown in prostate cancer that miR-1296 can decrease the expression of CDK2 by acting as a potential therapeutic target [22]. Yet how miR-1296 affects CDK2 expression in cervical cancer is largely unknown according to prior reports. We suggested that loss of miR-1296 elevated CDK2 expression, which curtailed apoptosis of cervical cancer cells. Specifically, Bax is known as a pro-apoptosis factor while Bcl-2 is one of the anti-apoptosis factors [23]. The experimental data showed that overexpression of CDK2 induced increased levels of Bcl-2, reflecting that circ_000520 mediated cell apoptosis through miR-1296/CDK2.

Further, the function of hsa_circ_0000520/miR-1296/CDK2 in cervical cancer was verified by performing in vivo experiments. The results displayed that silencing hsa_circ_0000520 or CDK2 reduced weight and volume of graft tumors in nude mice. Decreased volume and weight of tumors indicate cervical cancer is ameliorated [24]. Although no circRNA-based therapeutic candidate is currently applied in the clinical setting against cervical cancer, the present experimental data highlighted the significance of hsa_circ_0000520 in cervical cancer progression as a potential biomarker of prognosis and a therapeutic target. Therefore, we propose that targeting hsa_circ_0000520-miR-1296 axis is a potential strategy for treatment of cervical cancer.

## Conclusions

**Fig. 10:**
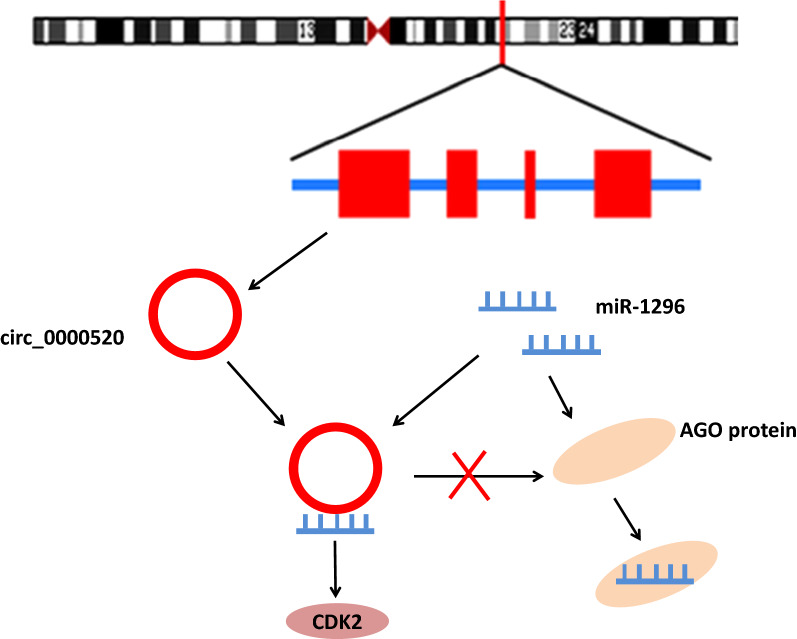
Mechanistic diagram depicting hsa_circ_0000520/miR-1296/CDK2 involvement in cervical cancer

In summary, our results indicated that hsa_circ_0000520 could inhibit the expression of miR-1296 to upregulate CDK2, which further accelerated the proliferation of cervical cancer cells, promoted cell cycle and suppressed cell apoptosis (Fig. 10). Our findings present a novel understanding of the molecular events implicated in pathogenesis of cervical cancer and offer a putative target for its treatment. However, an in-depth study of the circRNA–miRNA interaction network in cervical cancer is required to identify other relevant miRNAs regulated by hsa_circ_0000520, which may help to further elucidate the molecular mechanisms underlying cervical cancer progression. Furthermore, in future studies animal experiments performed using primary cells obtained from cervical cancer tissues can provide better translational evidence as compared to xenograft models generated using cell lines.

## Supplementary Information

**Additional file 1: Table S1.** Primer sequences used for RT-qPCR.

## Data Availability

Data sharing not applicable to this article as no datasets were generated or analyzed during the current study.

## Abbreviations

circRNA: Circular RNA
miRNAs: MicroRNAs
FIGO: Federation of Gynecology and Obstetrics
FBS: Fetal bovine serum
RIPA: Radio immunoprecipitation assay
ECL: Enhanced chemiluminescence
Mut: Mutant type
IHC: Immunohistochemistry
FISH: Fluorescence in situ hybridization
CCK-8: Cell counting kit-8
EdU: 5-Ethynyl-2’-deoxyuridine
RIP: RNA binding protein immunoprecipitation
SD: Standard deviation
ANOVA: Analysis of variance
