# Supplementary material for: Hsa_circ_0000520 overexpression increases CDK2 expression via miR-1296 to facilitate cervical cancer cell proliferation
Source: J Transl Med. 2021 Jul 20;19:314. doi: 10.1186/s12967-021-02953-9 (PMC8290540; doi:10.1186/s12967-021-02953-9)
Supplement: Supplementary file 1 — Additional file 1: Table S1. Primer sequences used for RT-qPCR. [file 12967_2021_2953_MOESM1_ESM.docx]

**Table S1** Primer sequences used for RT-qPCR.

|  | Primer sequences |
| --- | --- |
| hsa_circ_0000520 | F: 5'-GTCTGAGACTAGGGCCAGAGGC-3' |
|  | R: 5'-GACATGGGAGTGGAGTGACAGG-3' |
| RPPH1 | F: 5ʹ-GTCACTCCACTCCCATGTCC-3ʹ |
|  | R: 5ʹ-CAGCCATTGAACTCACTTCG-3ʹ |
| miR-1296 | F: 5'-TGGTGTCGTGGAGTCG-3' |
|  | R: 5'-CAGCAGCAATTCATGT-3' |
| CDK2 | F: 5'-AATCCGCCTGGACATGAGA-3' |
|  | R: 5'-TCCAGCTTGACAATATTAGGA-3' |
| U6 | F: 5'-GTACAAAATACGTGACGTAGAAAG-3' |
|  | R: 5'-GGTGTTTCGTCCTTCCAC-3' |
| GAPDH | F: 5'-ATGGAGAAGGCTGGGGCTC-3' |
|  | R: 5'-AAGTTGTCATGGATGACCTTG-3' |

Note: RT-qPCR, reverse transcription quantitative polymerase chain reaction; RPPH1, ribonuclease P RNA component H1; F, forward; R, reverse; miR, microRNA; CDK2, cyclin-dependent kinase 2; GAPDH, glyceraldehyde-3-phosphate dehydrogenase.
